# Thoracoscopic versus conventional thoracotomy for esophageal atresia/tracheoesophageal fistula repair: a comprehensive meta-analysis of 25 comparative studies

**DOI:** 10.1007/s00383-025-06182-9

**Published:** 2025-09-09

**Authors:** Amani N. Alansari, Marwa Messaoud, Salma Mani, Mohamed Sayed Zaazouee, Hanan Youssif, Amine Ksia

**Affiliations:** 1 Department of Pediatric Surgery, Hamad Medical Corporation, P.O Box: 3050, Doha, Qatar; 2 Pediatric Surgery Department, Fattouma Bourguiba University Hospital, Monastir, Tunisia; 3 Faculty of Medicine of Monastir, University of Monastir, Monastir, Tunisia; 4 Research Laboratory of Congenital Anomalies and Childhood Cancer LR12SP13, University of Monastir, Monastir, Tunisia; 5 Faculty of Medicine, Al-Azhar University, Assiut, Egypt; 6 Pediatric surgery department, Benghazi medical center, Benghazi district, Benghazi, Libya

**Keywords:** Esophageal atresia, Tracheoesophageal fistula, Thoracoscopic repair, Minimally invasive surgery, Thoracotomy, Meta-analysis

## Abstract

**Purpose:**

This meta-analysis compares thoracoscopic versus open thoracotomy repair of esophageal atresia with tracheoesophageal fistula (EA/TEF).

**Methods:**

We systematically searched PubMed, Web of Science, Cochrane Library, and Scopus from inception to April 2025 for studies comparing thoracoscopic versus conventional thoracotomy approaches. Two independent reviewers screened studies, extracted data, and assessed risk of bias using appropriate tools. Meta-analyses were conducted using RevMan 5.4 software.

**Results:**

A total of 25 studies (24 observational and one randomized controlled trial, including 3087 patients) were included. Thoracoscopic repair was associated with longer operative time (mean difference [MD] = 20.94 min; *p* = 0.005) but showed significant advantages in reducing mortality (risk ratio [RR] = 0.52; *p* = 0.01), musculoskeletal complications (RR = 0.08; *p* < 0.0001), and wound infections (RR = 0.21; *p* = 0.02). It also led to shorter ICU stays (MD = −1.09 days; *p* = 0.005) and earlier initiation of oral feeding (MD = −1.12 days; *p* = 0.02). However, the risk of anastomotic stricture requiring dilation was higher (RR = 1.54; *p* < 0.00001). No significant differences were found in anastomotic leak rates, recurrent fistula, respiratory complications, or need for fundoplication.

**Conclusions:**

Thoracoscopic repair of EA/TEF is associated with perioperative benefits over conventional thoracotomy, including significantly lower mortality and a markedly reduced incidence of musculoskeletal complications. However, this approach is associated with a higher risk of anastomotic stricture requiring dilation, and these differences may partly reflect patient selection factors.

**Supplementary Information:**

The online version contains supplementary material available at 10.1007/s00383-025-06182-9.

## Introduction

Esophageal atresia with tracheoesophageal fistula (EA/TEF) represents one of the most challenging congenital anomalies affecting approximately 1 in 3500–4500 live births worldwide [1, 2]. This condition, marked by esophageal discontinuity and often a tracheoesophageal fistula, necessitates surgical correction to restore esophageal continuity and prevent life-threatening complications [3]. For decades, conventional thoracotomy (TT) has been the gold standard surgical approach for repair, providing direct visualization and access to the thoracic cavity through a posterior lateral incision [4]. However, this approach is associated with notable morbidity, including musculoskeletal deformities, chest wall asymmetry, and considerable postoperative pain [5, 6].

The advent of minimally invasive techniques (MIT) has introduced an alternative approach that uses smaller incisions and provides better visualization, potentially reducing surgical trauma and improving recovery [7]. Despite these advantages, concerns remain regarding its technical complexity, longer operative times, and the potential for intraoperative complications such as hypercapnia due to CO₂ insufflation [8]. Comparative studies have yielded mixed results. For instance, Al Tokhais et al. (2008), in a case–control study, reported no notable difference in anastomotic leak rates between the two approaches [9], whereas Ma et al. (2012) observed a markedly prolonged operative time associated with the thoracoscopic technique [10]. Yang et al. (2021) noted an increased risk of stricture requiring dilation in the thoracoscopy (TS) group in a retrospective cohort study [11].

Thoracoscopic repair varies widely across pediatric centers, reflecting ongoing debate over its safety, efficacy and long-term outcome [12]. Such variability in practice patterns underscores the need for a comprehensive evaluation of the available evidence to guide clinical decision-making. Previous reviews have provided valuable insights but were limited by smaller sample sizes and evolving surgical techniques [6, 13].

This meta-analysis compares thoracoscopic and open TT repair of EA/TEF, focusing on key clinical outcomes. The aim is to provide evidence-based guidance for pediatric surgeons, healthcare providers, and families to support informed decision-making and optimize outcomes and resource use.

## Methods

This systematic review was performed following the Cochrane Handbook for Systematic Reviews of Interventions and is reported in line with the Preferred Reporting Items for Systematic Reviews and Meta-Analyses (PRISMA) guidelines [14, 15].

### Eligibility criteria

We included randomized controlled trials (RCTs) and observational studies (cohort and case–control studies) comparing thoracoscopic and conventional TT approaches for the repair of EA/TEF, with no restrictions on age at surgery, sex, or associated anomalies. We excluded case reports, case series, non-comparative studies, animal studies, non-English studies, and studies with fewer than 10 participants per group. Outcomes included operative time, postoperative complications (anastomotic leak, stricture requiring dilation, mortality, recurrent TEF), length of hospital stay, time to extubation, intensive care unit (ICU) stay duration, time to oral feeding, musculoskeletal and respiratory complications, wound infection, need for fundoplication, and arterial blood gas parameters [Potential of hydrogen (pH), partial pressure of carbon dioxide (PCO₂), end-tidal carbon dioxide (ETCO₂)].

### Information sources, search strategy, and study selection

We searched PubMed, Web of Science (WOS), Cochrane Library, and Scopus from inception to April 2025, with no language or date restrictions. The search strategy, developed per the Cochrane Handbook for Systematic Reviews of Interventions, used MeSH and free-text terms like “esophageal atresia,” “tracheoesophageal fistula,” “thoracoscopy,” and “thoracotomy” (Supplementary Table 1). We searched the reference lists of included studies and reviews. Two authors independently screened titles and abstracts, removed duplicates, and assessed full texts for eligibility. Disagreements were resolved via discussion or a third author.

### Data collection process

Two authors independently extracted data using a standardized form based on the Cochrane Handbook, capturing study characteristics (design, country, year), participant details (sample size, age, sex, birth weight, anomalies), intervention specifics (surgical technique, positioning, conversion rates), and outcomes. Follow-up durations and adverse events were noted. Any discrepancies were resolved through discussion.

### Risk of bias assessment

Two reviewers independently assessed the risk of bias for each study using tools suited to the study type. For cohort and case–control studies, the Newcastle–Ottawa Scale (NOS) was used to assess selection of participants, comparability of groups, and ascertainment of outcomes. These studies were rated as good, fair, or poor quality [16]. For randomized controlled trials, the Cochrane Risk of Bias Tool was used, evaluating domains including random sequence generation, allocation concealment, blinding, incomplete outcome data, and selective reporting [17]. Disagreements in the quality assessments were resolved through discussion.

### Statistical analysis

Meta-analyses were conducted using Review Manager (RevMan) version 5.4. For continuous outcomes, we calculated mean differences (MD) with 95% confidence intervals (CI); for dichotomous outcomes, we calculated risk ratios (RR) with 95% CI. When means and standard deviations were not directly available, they were estimated using medians, ranges, and interquartile ranges as per Cochrane guidelines.

Heterogeneity was evaluated using the Chi-squared (χ^2^) test and quantified with the I^2^ statistic, with a p-value below 0.10 indicating the presence of significant heterogeneity. Fixed-effect model was used for analyses with low heterogeneity; random-effects model was applied when substantial heterogeneity was detected. Sensitivity analyses excluded outliers. Statistical significance was set at p < 0.05.

## Results

**Fig. 1:**
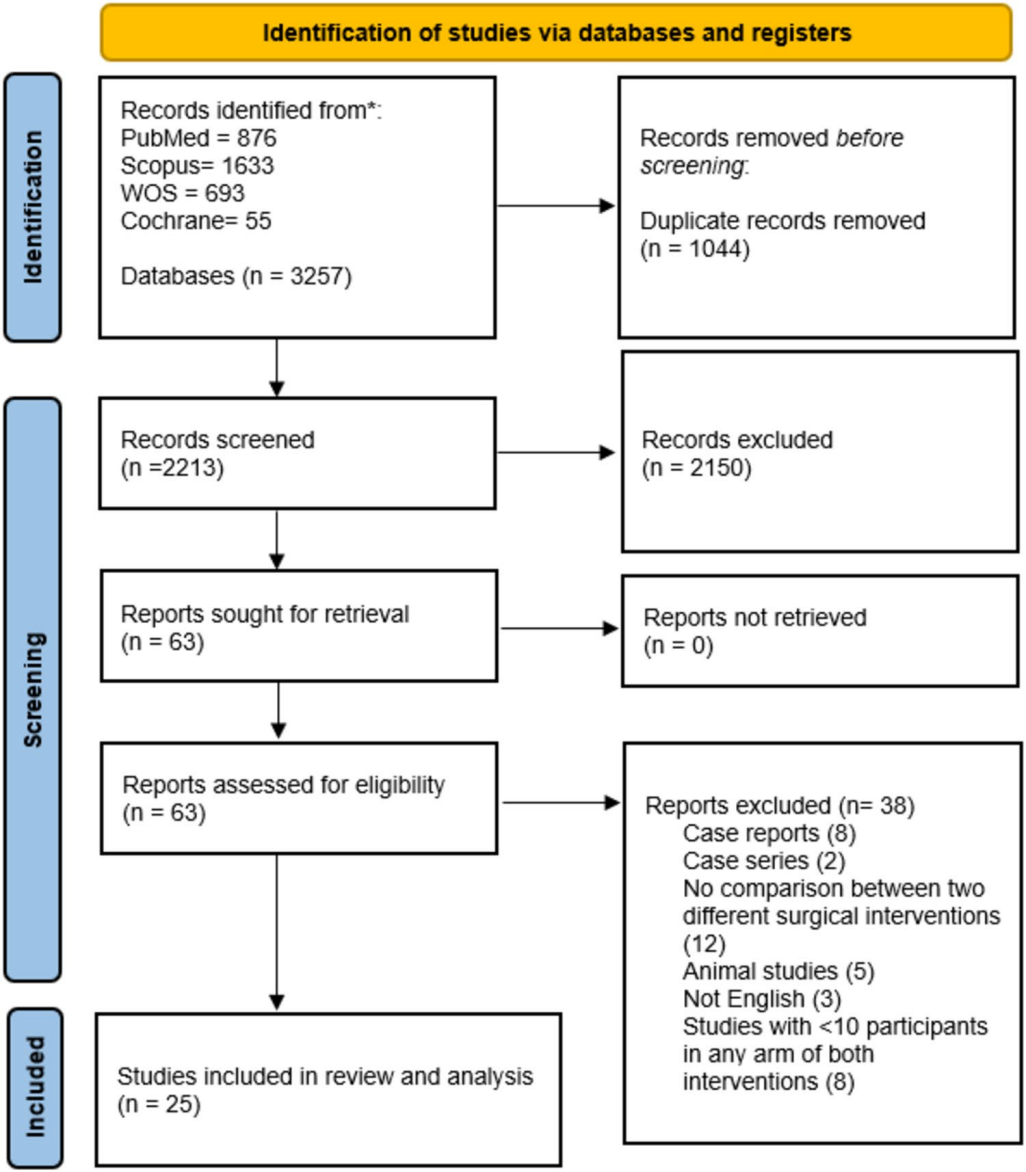
PRISMA flow diagram of study selection

A total of 3257 records were identified through four major databases, with 1044 duplicates removed before screening. Of the 2213 records screened, 2150 were excluded based on title and abstract. 63 full-text articles were assessed for eligibility, of which 38 were excluded for reasons such as case reports, non-comparative designs, animal studies, or insufficient sample size. Ultimately, 25 studies met the inclusion criteria [5, 8–11, 18–37] (Fig. 1).

### Baseline and characteristics of the included studies

The included studies, conducted across various countries such as the USA, China, Italy, and others, primarily employed retrospective cohort designs, with one randomized comparative study [25], and one case–control study [9]. Thoracoscopic repair offers a minimally invasive approach, while TT—muscle-sparing or extrapleural—involves open access. Associated anomalies, mainly cardiovascular, gastrointestinal, genitourinary, and syndromes like VACTERL, were frequently reported. Baseline characteristics showed sample sizes ranging from 11 to 217 in the TS group and 14–538 in the TT group. The median age at surgery ranged from 1 to 7.3 days, with male predominance in both groups. Birth weights varied between 2.28 kg and 3.30 kg. Follow-up durations extended up to 15 years. Conversion to open surgery differed widely across studies, from 0 to 22 (27.5%) cases. Further details are shown in Tables 1, 2*.*

**Table 1:** Overview of included studies comparing thoracoscopic and open repair of esophageal atresia with tracheoesophageal fistula

ID	Country	Study design	Laparoscopic surgery approach details	Open surgery approach details	Type of EA	Associated anomalies	Main results
Hyman 2025	USA	Retrospective cohort study	Thoracoscopic repair	Right-sided thoracotomy repair	Type C	NR	Thoracoscopic repair had higher rates of anastomotic stricture and recurrent laryngeal nerve injury compared to open surgery, with only slight benefit in reduced opioid use
Datta 2025	USA	Retrospective cohort study	Trans-pleural (thoracoscopic repairs	Extra-pleural or trans-pleural	Type C	Small for gestational age < 10th percentile Ventricular septal defect VACTERL association	Clinical and operative factors linked to esophageal anastomotic leaks after type C repair in infants with inter-center variability suggesting that identifying best practices could enhance patient care
Borselle 2024	Poland	Retrospective two-center cohort study	Right-sided thoracoscopy; azygos vein preserved; blunt dissection without electrosurgery; 3–5 mm ports around scapula; multi-staged repairs for long-gap EA (internal traction technique for types A/B)	Open right posterolateral thoracotomy via 4th or 5th intercostal space, with or without muscle-sparing technique; azygos vein ligated; chest drain routinely placed; multi-stage repair if primary anastomosis not possible	Types A, B, C, D (mostly type C)	32.4% in thoracoscopy group and 27.3% in thoracotomy group	The thoracoscopy group had fewer musculoskeletal complications: scoliosis (1.5% vs 13.6%), rib fusion (0% vs 37.1%), and combined deformities (0% vs 9.1%); No cases of scoliosis exceeding 20° were observed in the thoracoscopy group, and the incidence of reoperations following thoracoscopic procedures was significantly lower compared to open approaches. This suggests that thoracoscopy not only minimizes the risk of severe postoperative spinal deformity but also reduces the likelihood of requiring additional surgical interventions
Fabrizio 2024	Italy	Retrospective cohort study	5 mm trocar below right scapula tip + two additional trocars (3 mm and 5 mm) Semi-prone position TEF closed with clips End-to-end esophago-esophageal anastomosis with trans-anastomotic tube Chest drain placed through posterior trocar site	Right 5th intercostal space thoracotomy Intrapleural access TEF ligated and divided with sutures End-to-end esophago-esophageal anastomosis with interrupted sutures Trans-anastomotic orogastric tube and para-anastomotic chest drain placed	Type C	Cardiovascular, gastrointestinal, anorectal, urogenital, limb, skeletal, respiratory, CNS, and others (e.g., VACTERL, CHARGE syndrome)	Thoracoscopic repair resulted in better QoL in terms of physical and psychosocial health compared to thoracotomy and was assessed with a mean follow-up period of 7.3 ± 2.5 years for the thoracotomy group and 4.9 ± 1.7 years for the thoracoscopy group
Mangray 2024	South Africa	Retrospective cohort study	Three-port thoracoscopy (5 mm camera, 3 mm working ports), CO₂ insufflation (4–6 mmHg), bronchial blocker for larger infants	Posterolateral thoracotomy	Type C, Type A Type E	NR	Thoracoscopic repair showed comparable outcomes to open repair, with lower mortality (5.5% vs. 20.5%) but no significant differences in complications
Yalcin 2024	USA	Retrospective cohort study	Three-port thoracoscopy with CO₂ insufflation, prone position, fistula ligation, and interrupted sutures for anastomosis	Posterolateral thoracotomy (standard/muscle-sparing), same anastomosis technique as thoracoscopy	Type C and D	VACTERL anomalies, complex cardiac anomalies, vascular pathologies, duodenal atresia, syndromes/chromosomal abnormalities	Thoracoscopic repair showed shorter ventilation and hospital stay but higher anastomotic stricture rates compared to open repair. Long-term tube feeding needs were lower in the thoracoscopy group
Aslan 2023	Turkey	Retrospective cohort study	3 trocars (1 × 4 mm camera, 2 × 3 mm ports); CO₂ insufflation; azygos vein division; TEF ligation and esophageal anastomosis with 5/0 Polydioxanone sutures	Muscle-sparing posterolateral incision (4th/5th intercostal space); extra-pleural dissection; azygos vein ligation; TEF ligation and esophageal anastomosis with 5/0 Polyglactin sutures	Type C	Cardiovascular (most common), gastrointestinal, genitourinary, trisomy 18	No significant differences in complication rates, mortality, or long-term outcomes between thoracoscopy and thoracotomy groups. Thoracoscopy had shorter gavage feeding initiation but longer surgery time
Zou 2023	China	Retrospective cohort study	Left semi-prone position, 3 trocars (5 mm + two 3 mm), CO₂ insufflation (6–8 mmHg)	Left lateral position, right posterolateral thoracotomy (4th intercostal space)	Type C	Cardiovascular (23.1%), gastrointestinal (6.1%), genitourinary (3.3%), skeletal (9.2%), VACTERL association (33.1%)	Thoracoscopic repair for congenital EA is safe and has similar perioperative and mid-term outcomes to open surgery
Thakkar 2021	UK	Retrospective cohort study	Thoracoscopic repair with 3-mm set, semi-prone/lateral position, insufflation pressures of 6–8 mmHg	Open repair via thoracotomy with extra-pleural dissection	Type C	Cardiac co-morbidities (59% in open group, 43% in the thoracoscopic group)	Thoracoscopic repair had a higher stricture rate (72%) compared to open (43%), but similar dilation requirements
Yang 2021	China	Retrospective cohort study	Minimally invasive thoracoscopic repair with variations in fistula closure (ligation, Hem-o-lok clip)	Open thoracotomy with primary fistula closure (94.31% ligation)	Type C	158 patients had other congenital diseases (no syndromic, VACTERL syndrome, syndromic diagnosis)	Thoracoscopic surgery for Gross type C is safe and effective, with comparable operative time and postoperative complications to thoracotomy
Zhang 2020	China	Retrospective cohort study	Three small incisions (0.5 cm and two 0.3 cm), thoracoscopic ligation of fistula and end-to-end esophageal anastomosis, chest tube insertion	Traditional open surgery via 5th intercostal posterolateral incision, direct ligation of the fistula and esophageal anastomosis	Type C	Congenital anal atresia, Uro-nephrosis	Thoracoscopic surgery for type C esophageal atresia shows comparable safety and effectiveness to traditional surgery with advantages such as smaller incision, reduced chest wall deformity, and faster recovery
Elhattab 2020	France	Retrospective cohort study	Left lateral or semi-prone position, 3–4 trocars, CO₂ insufflation (5 mmHg), azygos vein division, esophageal anastomosis with absorbable sutures, chest drain insertion	Left lateral position, right poster-lateral thoracotomy via 4th or 5th intercostal space, azygos vein division, esophageal anastomosis with absorbable or non-absorbable sutures, chest drain insertion	Type C	Cardiac (24% TS, 28% TT), others (47% TS, 48% TT)	Thoracoscopic repair had comparable complications to thoracotomy, shorter ventilation & hospital stay, but longer operative time and higher CO₂ levels
Khodary 2019	Egypt	Randomized comparative study (non-blinded)	Prone position, 5 mm and 3 mm ports, CO₂ insufflation, sparing azygos vein, single-layer interrupted sutures	Right lateral thoracotomy, muscle-sparing, azygos vein ligated in 66.66%, single-layer interrupted sutures	Type C	Cardiac (83.33%), Renal (13.33%), Anorectal (6.66%), Down syndrome (6.66%)	Thoracoscopic repair is safe, effective, and offers better cosmetic outcomes and azygos vein preservation compared to open repair, with similar complication rates
Zani 2017	Canada	Retrospective cohort study	Thoracoscopic repair with CO₂ insufflation	Open surgery via laparotomy (CDH) and thoracotomy (EA/TEF)	Type C	NR	Thoracoscopic repair of EA/TEF and CDH is associated with more severe intraoperative acidosis compared to open surgery, but without clear impact on early postoperative outcomes
Fusco 2017	USA	Retrospective cohort study	Minimally invasive thoracoscopic repair	Open thoracotomy (muscle-sparing and non-muscle-sparing)	Type C (87%), Type A (6%), Type D (4%), Type E (3%)	VACTERL association, sepsis, prematurity, respiratory failure (specific % not given)	TAT use associated with higher stricture rate (56% vs. 17%, p < 0.0005); no difference in leak rate (p = 0.27). Thoracoscopy showed fewer musculoskeletal complications (0% vs. 28% in open group)
Nice 2016	USA	Retrospective cohort study	Thoracoscopic repair,	Open thoracotomy	Types C (84%), A, B, D, E (remaining)	Cardiovascular (45.5%), GU, CNS, vertebral anomalies	Thoracoscopy and staged repairs increased stricture risk; cardiovascular anomalies protective
Woo 2015	USA	Retrospective cohort study	Thoracoscopic esophageal mobilization, no electrocautery used at proximal pouch to minimize nerve injury	Traditional open thoracotomy with ligation of fistula and esophageal anastomosis	Type C (majority), plus pure EA cases	Cardiac anomalies common in VCP group	Thoracoscopic group had higher vocal cord paresis (52%) compared to open group (7%)
Yamoto 2014	Japan	Retrospective cohort study	Semi prone position, CO₂ insufflation (4–8 mmHg), preserved azygos vein, intracorporeal suturing	Lateral thoracotomy, ligated azygos vein, similar anastomosis technique	Type C	54.5% TR group vs. 46.7% OR group had anomalies	Thoracoscopic repair is feasible and safe in selected cases > 2 kg birth weight without severe anomalies
Koga 2014	Japan	Retrospective cohort study	Thoracoscopic repair with CO₂ insufflation, semi-prone position, 3–5 mm cannulas	Open lateral thoracotomy (intra- or extra-pleural)	Type C	37% of cases (48% in TR group, 30% in OR group)	TR resulted in fewer respiratory complications, less narcotic use, and shorter hospitalization compared to OR
Ma 2012	China	Prospective cohort study	CO₂ insufflation (2–6 mmHg), three-quarters prone position, pressure-control ventilation	Conventional thoracotomy, same anesthetic management as TR group	Type C	Patent ductus arteriosus, anorectal atresia, VSD/ASD, right aortic arch, bronchopulmonary dysplasia	TR is safe and tolerable with shorter extubating time compared to open repair, despite longer operative time and transient hypercarbia during TR
Burford 2011	USA	Retrospective cohort study	Thoracoscopic repair (multi-institutional data)	Right thoracotomy (71 patients), left thoracotomy (1 patient), muscle-sparing technique (16 patients)	Short-gap esophageal atresia	61% had significant anomalies, including cardiac defects (21%), VACTERL syndrome (17%), duodenal atresia (2.7%), trisomy 18 (2.7%), and Down syndrome (1.3%)	Thoracotomy and thoracoscopic repair had similar complication rates, but thoracotomy showed trends toward fewer anastomotic leaks (2.7% vs. 7.6%) and lower need for fundoplication (12% vs. 24%). No significant musculoskeletal sequelae were attributed to thoracotomy
Ceelie 2011	Netherlands	Retrospective cohort study	Thoracoscopic repair of esophageal atresia and congenital diaphragmatic hernia	Conventional open surgery for esophageal atresia and congenital diaphragmatic hernia	NR	CDH was also studied alongside EA	No significant difference in postoperative opioid consumption between MAS and conventional surgery for EA or CDH in neonates
Szavay 2011	Germany	Retrospective cohort study	Three-port thoracoscopy with CO₂ insufflation (4–6 mmHg), fistula ligation, and end-to-end anastomosis	Lateral thoracotomy with extra-pleural access, fistula ligation, and anastomosis		47% of all patients (e.g., VACTERL association, cardiac defects, others)	Thoracoscopic repair had comparable perioperative outcomes to open surgery, with longer operative time and higher intraoperative pCO₂ but no difference in complications or postoperative recovery
AlTokhais 2008	Saudi Arabia	Retrospective multi-institutional case–control matched study	Trans-pleural thoracoscopy with 3 ports, Ligature sealing of azygos vein, intracorporeal sutures for anastomosis	Muscle-cutting right posterolateral extra-pleural thoracotomy via the fourth interspace, single-layer anastomosis	Mostly Type C; 2 cases of Type A in thoracoscopic group	Cardiac anomalies (39.1% in TR group vs 59.1% in COR group)	TR of tracheoesophageal fistulas is safe and shows comparable outcomes to conventional open repair regarding operative time, leak rates, and stricture development
Miyano 2007	Japan	Retrospective cohort study	Thoracoscopic esophago-esophagostomy, no conversions, some cases required fundoplication for GERD	Open esophago-esophagostomy via thoracotomy, some cases required fundoplication for GERD	Type C	OR group: Anorectal malformation (1), Ileum atresia (1), Trisomy 21 (1) TR group: Anorectal malformation (3), Duodenal atresia (2), Trisomy 21 (2)	QoL scores were initially lower in thoracoscopic repair but improved over time to match those of open repair by school age, with no conversions to open surgery. QOL was evaluated both in the early postoperative period (1 year) and in the longer term, extending to school age (approximately 6 years or more)

*EA* esophageal atresia, *TEF* tracheoesophageal fistula, *USA* United states of America, *CDH* congenital diaphragmatic hernia, *GERD* gastroesophageal reflux disease, *TR* thoracoscopic repair, *VACTERL association* stands for vertebral defects, anal atresia, cardiac defects, tracheo-esophageal fistula, renal anomalies, and limb abnormalities, *TAT* trans-anastomotic feeding tube, *COR* conventional open repair, *QOL* quality of life, *MAS* minimally access surgery, *CHARG* stands for coloboma, heart defects, atresia choanae, growth retardation, genital abnormalities, and ear abnormalities, *CNS* central nervous system, *VSD* ventricular septal defect, *ASD* atrial septal defect, *VCP* vocal cord paralysis, *OR* operative repair, *CO2* carbon dioxide, *NR* not reported, *GU* genitourinary *TS* thoracoscopy, *TT* thoracotomy, *UK* United Kingdom

**Table:** Baseline characteristics of patients in included studies comparing thoracoscopic and open repair of esophageal atresia with tracheoesophageal fistula

ID	Group	Sample size	Mean age at surgery	Sex	Average birth weight (kg)	Follow-up period	Number of cases converted to open surgery
Hyman 2025	Thoracoscopy	80	1.44 days	63% male	3 kg (IQR 2.4–3.4)	NR	22
	Thoracotomy	538	1.33 days	59% male	2.7 kg		
Datta 2025	Thoracoscopy	79	NR	214 males, 151 females	2.7 kg [IQR 2.01–3.01]	120 days	
	Thoracotomy	271					
Borselle 2024	Thoracoscopy	68	NR	42.6% female, 57.4% male	2.5 ± 0.6 kg	6 years	
	Thoracotomy	44		47.7% female, 52.3% male	2.5 ± 0.8 kg	5.5 years	
Fabrizio 2024	Thoracoscopy	15	NR	9 males (60%), 6 females	2.475 kg	4.9 ± 1.7 years	
	Thoracotomy	17		9 males (53%), 8 females	2.385 kg	7.3 ± 2.5 years	
Mangray 2024	Thoracoscopy	18	2–38	NR	3.55 kg	NR	2
	Thoracotomy	39	1–24	NR	2.5 kg		
Yalcin 2024	Thoracoscopy	49	Similar in both groups	Male: 28, Female: 21	2.9 [IQR: 2.53–3.07] kg	3 years	21
	Thoracotomy	55		Male: 30, Female: 25	2.32 [IQR: 1.8–23] kg		
Aslan 2023	Thoracoscopy	14	2 days (median)	9 male, 5 female	2.675 kg	NR	
	Thoracotomy	31		17 male, 14 female	2.82 kg		
Zou 2023	Thoracoscopy	217	4 days	62.7% male	2.9	23.7 months (IQR 6.4–44.0 months)	
	Thoracotomy	142		66.2% male	2.73 kg		
Thakkar 2021	Thoracoscopy	34	NR	NR	3.3 (range: 1.79–4.2) kg	60 months (range: 2–120 months)	11
	Thoracotomy	61			2.28 (0.68–3.57) kg		
Yang 2021	Thoracoscopy	62	4 days (median)	44 boys, 18 girls	2.9 (2.673–3.2) kg	68 months (range: 22–117 months)	6
	Thoracotomy	128		88 boys, 40 girls	3 (2.5–3.26) kg		
Zhang 2020	Thoracoscopy	49	1.8 ± 0.6 days	28 boys / 21 girls	2887 ± 557 g	NR	
	Thoracotomy	43	1.6 ± 0.7 days	24 boys / 19 girls	2.66 ± 0.612 kg		
Elhattab 2020	Thoracoscopy	47	1 day (0–29 days)	NR	2.78 ± 0.65 kg	1 year	4
	Thoracotomy	140	1 day (0–7 days)		2.4 ± 0.65 kg		
Khodary 2019	Thoracoscopy	15	7.2 ± 5.90 days	8 male, 7 female	2.9 ± 0.306. kg	6 months	1
	Thoracotomy	15	6.33 ± 3.71 days	9 male, 6 female	3 ± 0.41 kg		
Zani 2017	Thoracoscopy	14	2 (1–3)	Male: Female = 6:8	3.06 ± 0.6 kg	46 months	5
	Thoracotomy	62	2 (1–4)	Male: Female = 37:25	2.6 ± 0.76 kg		
Fusco 2017	Thoracoscopy	16	2.3–2.4 days	NR	2.7 kg	9.62 years	10
	Thoracotomy	94				9.41 years	
Nice 2016	Thoracoscopy	20	NR	NR	2.8 kg	NR	3
	Thoracotomy	98			2.3 kg		
Woo 2015	Thoracoscopy	17	42 days	5 females, 12 males	2.8 kg	6 years	5
	Thoracotomy	14	42 days	4 females, 10 males	2.08 kg		
Yamoto 2014	Thoracoscopy	11	1.9 days (range 0–7)	7 male, 4 female	2.6 kg	at least 1 year	0
	Thoracotomy	15	3.7 days (range 0–10)	11 male, 4 female	2.7 kg		
Koga 2014	Thoracoscopy	25	3.1 ± 2.3 days	NR	2.6 ± 0.4 kg	NR	
	Thoracotomy	40	3.8 ± 2.9 days				
Ma 2012	Thoracoscopy	18	NR	Male: Female = 15/3	2.6 ± 0.8 kg	NR	2
	Thoracotomy	15		Male: Female = 7/8	2.3 ± 0.6 kg		
Burford 2011	Thoracoscopy	104	1.2 days	NR	2.6 kg	15 years	
	Thoracotomy	72	3.7 days		2.68 kg		
Ceelie 2011	Thoracoscopy	14	2 days	7 male/7 female	2.62 kg	7 days	1
	Thoracotomy	28	1 day	14 male/14 female	2.79 kg		
Szavay 2011	Thoracoscopy	25	1 day	36 girls, 32 boys	2.72 kg	NR	8
	Thoracotomy	32			2.09 kg		
AlTokhais 2008	Thoracoscopy	23	NR	NR	2.7 (± 0.74)kg	14.4 months (range 6–46 months)	3
	Thoracotomy	22			2.427 (± 0.726) kg	29.8 months (range 6–119 months)	
Miyano 2007	Thoracoscopy	13	7.3 days (range 2–30)	5 males/8 females	2.7 kg (range 2.1–3.8 kg)	6 years	0
	Thoracotomy	24	4.1 days (range 0–27)	16 males/8 females	2.6 kg		

*NR* not reported, *IQR* interquartile range

### Quality assessment

Overall, most cohort studies were found to be of good methodological quality, demonstrating consistency across key domains such as selection of cohorts, comparability of groups, and adequacy of outcome assessment. Mangray (2024), Woo (2015), and Burford (2011) were rated as fair, primarily due to concerns related to follow-up duration or exposure ascertainment [5, 27, 32] Supplementary Table 2.

The only case–control study, conducted by Al Tokhais (2008), also received a fair rating, with limitations noted in participant selection and group comparability [9] Supplementary Table 3.

The RCT by Khodary (2019) was assessed as having low risk of bias in random sequence generation, attrition, reporting, and other potential sources of bias. However, concerns were noted regarding allocation concealment and blinding of outcome assessment, which were rated as unclear, while blinding of participants was assessed as high risk [25] Supplementary Table 4.

### Operative outcomes

**Fig. 2:**
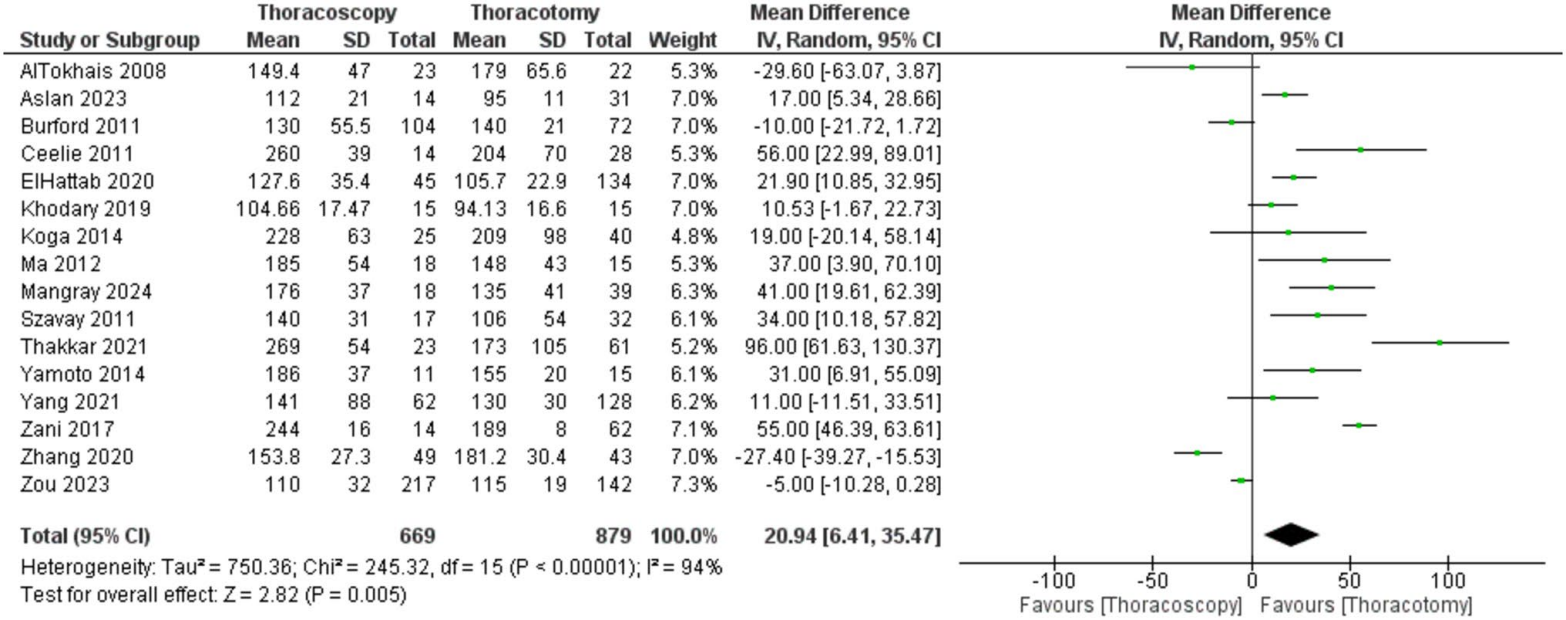
Forest plot of operative time (minutes)

The pooled analysis of 16 studies revealed that operative time was significantly longer in the TS group compared to the TT group, with a mean difference of 20.94 min (6.41 to 35.47; P = 0.005). However, significant and unresolvable heterogeneity was observed across the included studies (P < 0.00001; I^2^ = 94%) (Fig.2).

**Fig. 3:**
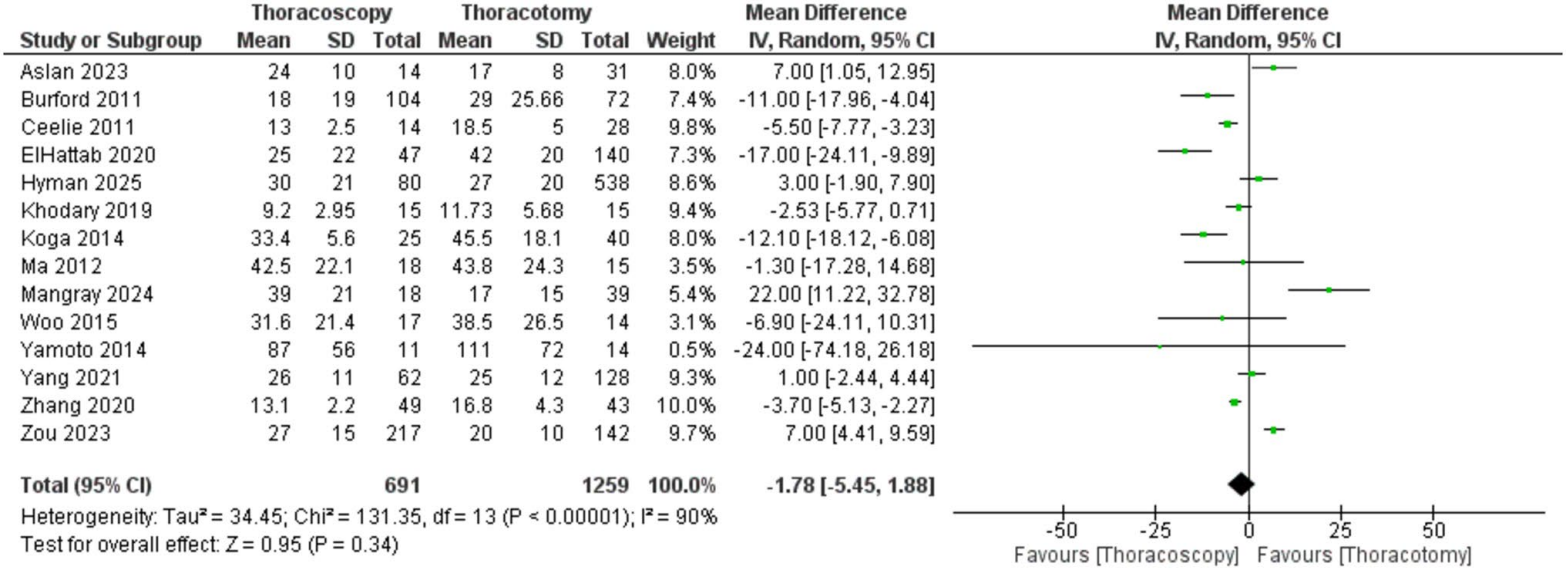
Forest plot of length of hospital stay (days)

Regarding hospital stay duration, the meta-analysis of 14 studies demonstrated no significant difference between the TS and TT groups (MD = −1.78 days; −5.45 to 1.88; P = 0.34), and the analysis revealed unresolvable heterogeneity among the included studies (P < 0.00001; I^2^ = 90%) (Fig.3).

**Fig. 4:**
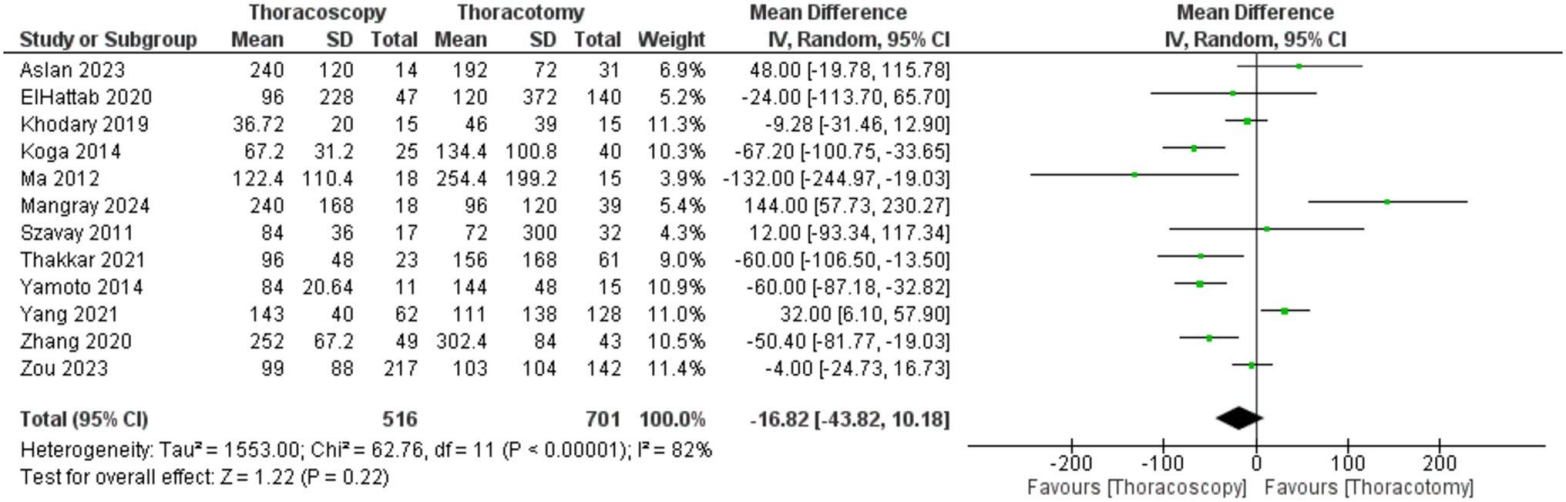
Forest plot of time to extubation (hours)

Also, the pooled analysis of 12 studies showed no meaningful difference in the time to extubation between the TS and TT groups. (MD = −16.82 h [−43.82, 10.18], P = 0.22), with unresolvable heterogeneity in the data (P < 0.00001; I^2^ = 82%) (Fig.4).

**Fig. 5:**
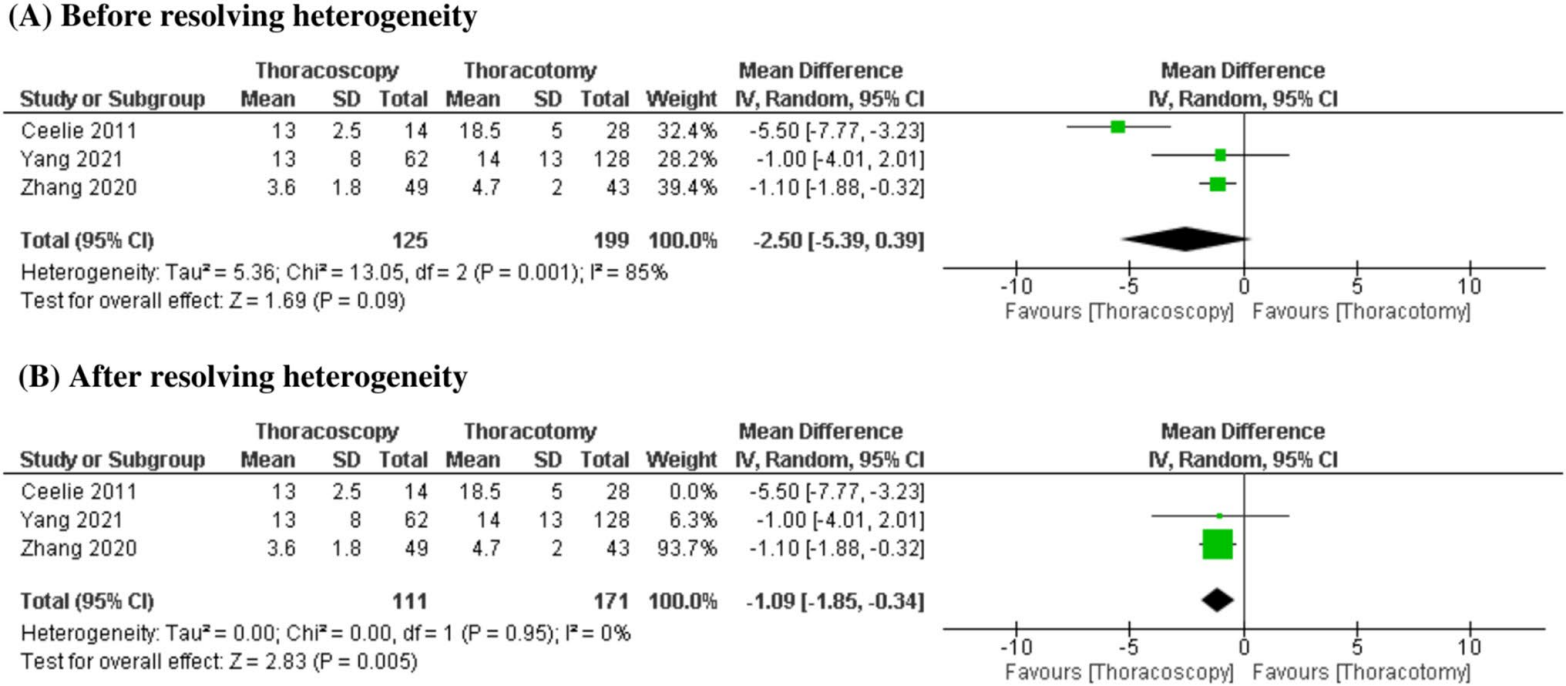
Forest plot of ICU stay duration (days)

Moreover, the pooled analysis of 3 studies showed an insignificant difference in ICU stay duration between the both groups (MD = 2.50 days [−5.39, 0.39], P = 0.09), with existing heterogeneity in the data (P = 0.001; I^2^ = 85%). After resolving heterogeneity by excluding Ceelie (2011) (P = 0.95; I^2^ = 0), TS was associated with a significantly lower ICU stay. (MD = −1.09 [−1.85, −0.34], P = 0.005) (Fig.5).

**Fig. 6:**
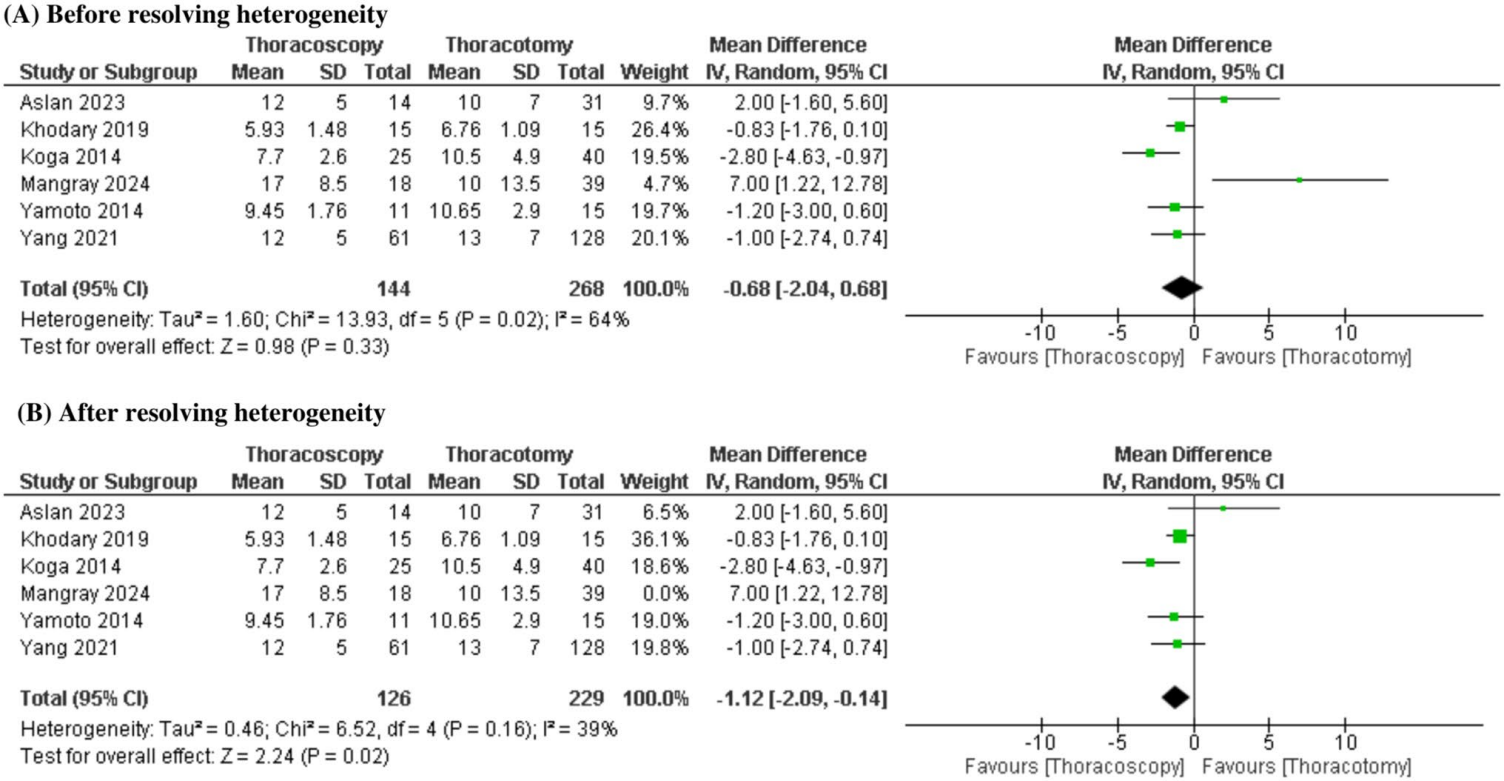
Forest plot of time to oral feeding (days)

Similarly, the analysis of 6 studies showed an insignificant difference in time to oral feeding between both groups (MD = −0.68 days [−2.04, 0.68], P = 0.33), but the data exhibited moderate heterogeneity (P = 0.02; I^2^ = 64%). After resolving heterogeneity by excluding (Mangray 2024) (P = 0.16; I^2^ = 39%), the pooled data showed a shorter time to oral feeding in the TS group compared to the TT (MD = −1.12 days [−2.09, −0.14], P = 0.02) (Fig.6).

### Postoperative complications

**Fig. 7:**
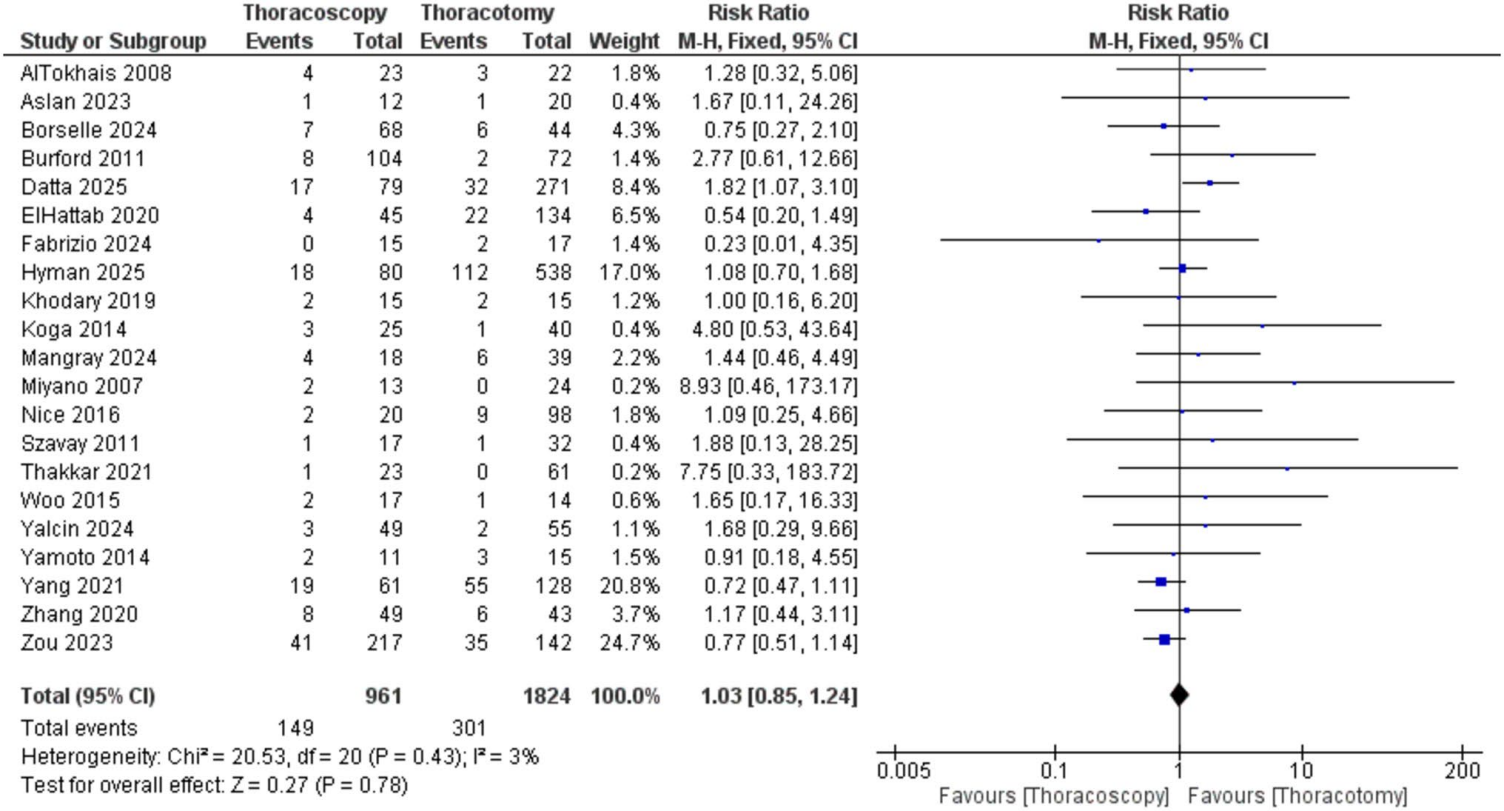
Forest plot of anastomotic leak

An analysis combining 21 studies revealed no anastomosis leak between the two groups (RR = 1.03 [0.85, 1.24], P = 0.78). The data showed low heterogeneity (P = 43; I^2^ = 3%) (Fig.7).

**Fig. 8:**
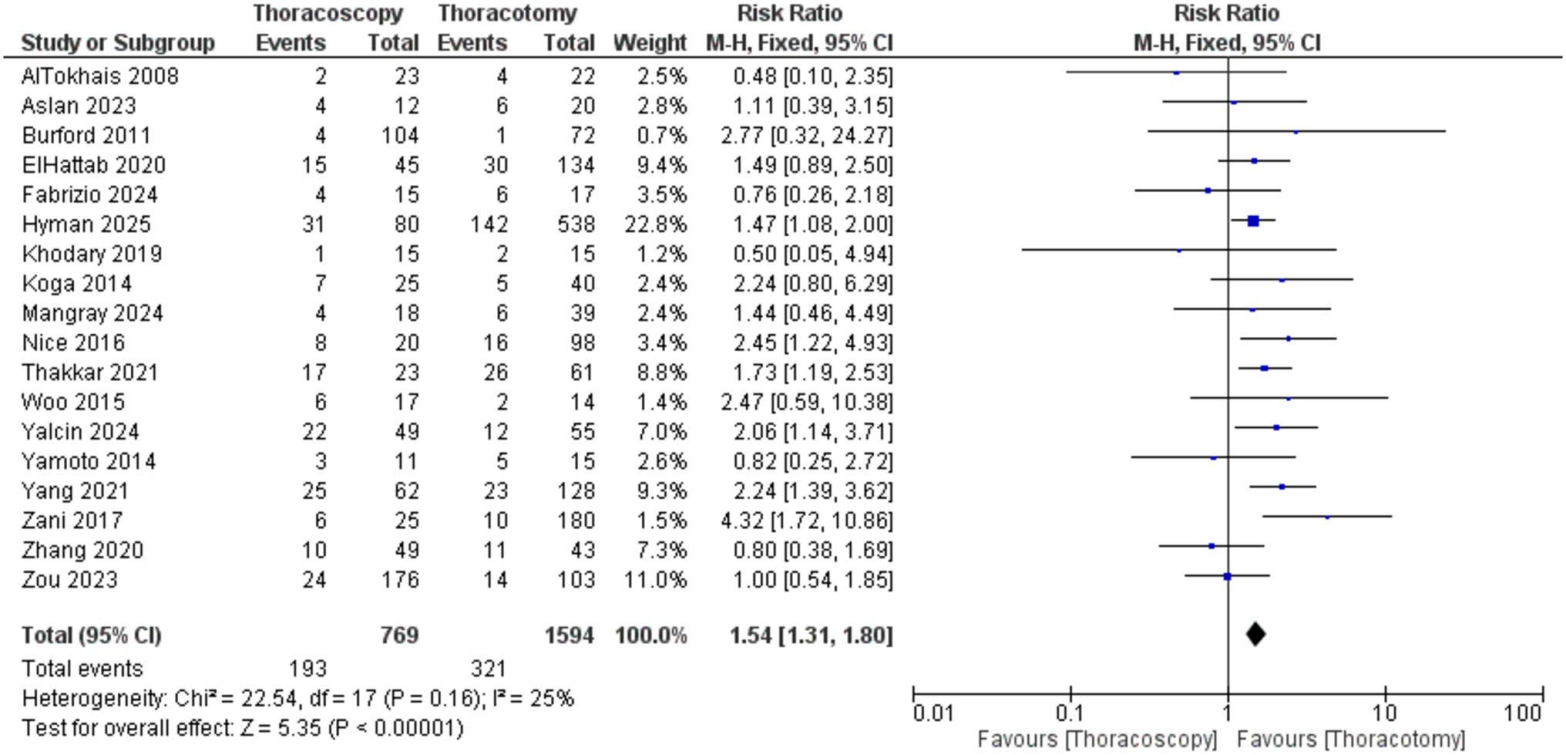
Forest plot of stricture requiring dilation

Regarding the risk of stricture (requiring dilation), a meta-analysis of 18 studies demonstrated a statistically significant increase in the TS group compared to the TT group (RR = 1.54 [1.31, 1.80], P < 0.00001), and the heterogeneity was low (P = 0.16; I^2^ = 25%) (Fig.8).

**Fig. 9:**
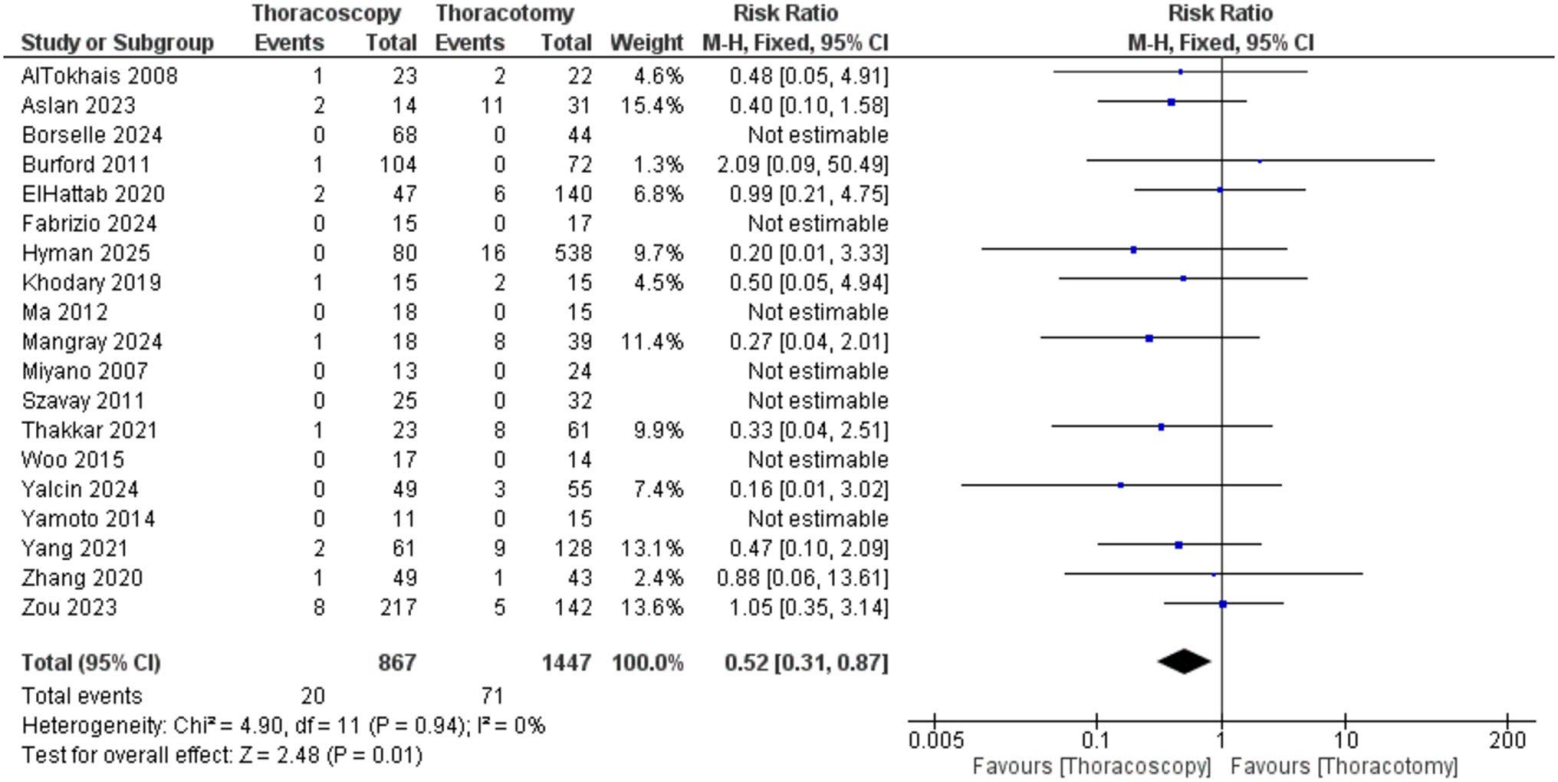
Forest plot of mortality rate

Also, meta-analysis of 12 studies demonstrated a statistically lower mortality rate among patients undergoing TS compared to those treated with TT (RR = 0.52 [0.31, 0.87], P = 0.01). No heterogeneity was observed across studies (P = 0.94; I^2^ = 0%). Seven studies reported no events in either group (Fig.9).

**Fig. 10:**
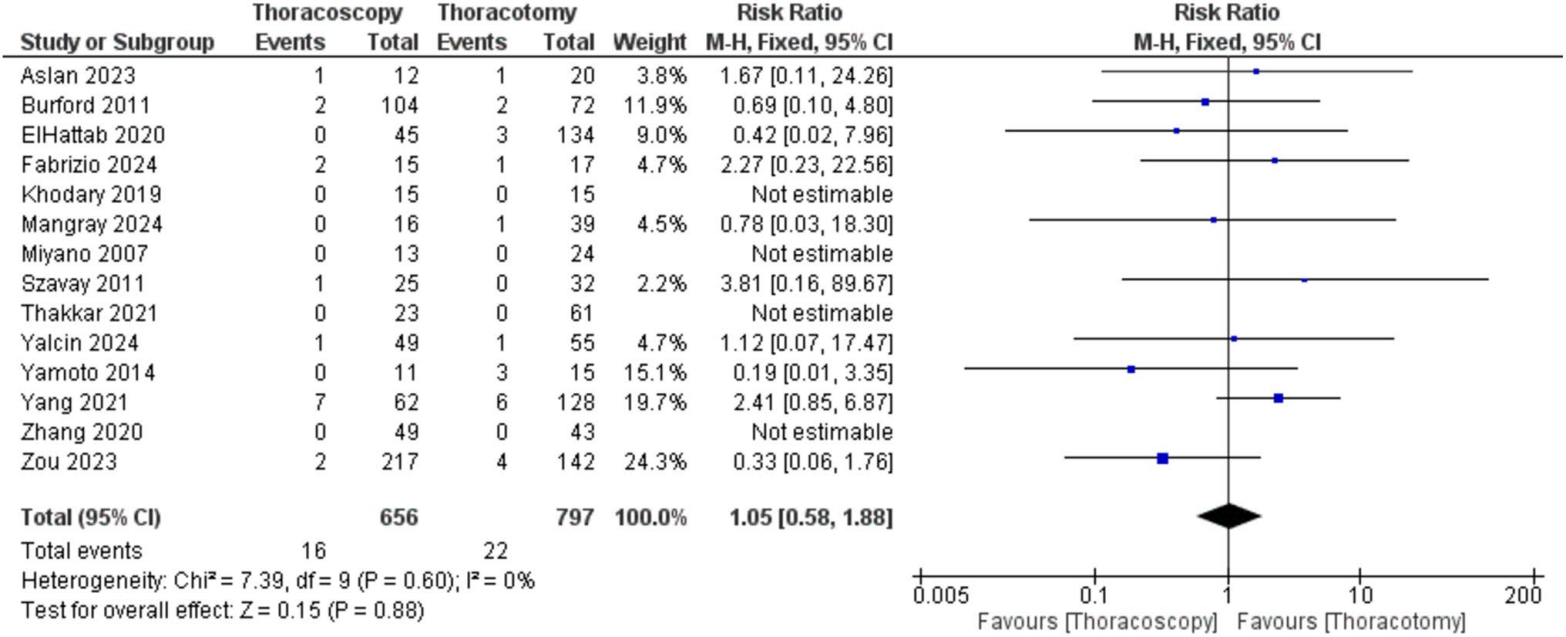
Forest plot of recurrent TEF

Regarding recurrent TEF rates, the pooled analysis of 10 studies showed no difference between TS and TT (RR = 1.05 [0.58, 1.88]; P = 0.88), with no heterogeneity detected (P = 0.60; I^2^ = 0%). Four studies reported no events in either group (Fig.10).

Regarding musculoskeletal complications, the analysis of four studies demonstrated a markedly lower incidence of musculoskeletal complications in the TS group (RR = 0.08 [0.02, 0.28]; P < 0.0001), with no observed heterogeneity (P = 0.68; I^2^ = 0%). One study reported no events in either group Supplementary Fig. 1.

Eleven studies reported no difference in respiratory complication rates between TS and TT (RR = 0.90 [0.49, 1.62]; P = 0.72), though substantial heterogeneity remained (I^2^ = 62%; P = 0.004) Supplementary Fig. 2.

Four studies showed lower wound infection rates with TS (RR = 0.21 [0.06, 0.76]; P = 0.02), with no heterogeneity (I^2^ = 0%; P = 0.81) Supplementary Fig. 3.

Eleven studies found no significant difference in subsequent fundoplication rates between TS and TT (RR = 1.16 [0.79, 1.70]; P = 0.44), with low heterogeneity (I^2^ = 29%; P = 0.17). One study reported no events in either group Supplementary Fig. 4.

### Physiological parameters

The pooled analysis collectively demonstrated no significant differences in physiological parameters between the TS and TT groups, including pH (MD = −0.04 [−0.09, 0.001], P = 0.08; I^2^ = 54%, 2 studies), PCO2 (MD = 3.92 [−2.84, 10.69], P = 0.26; I^2^ = 63%, 2 studies), and ETCO2 (MD = 3.51 [−5.00, 12.02], P = 0.42; I^2^ = 94%, 2 studies). Pooled analysis of two studies showed no significant differences between TS and TT in physiological parameters: pH (MD = −0.04 [−0.09, 0.001]; P = 0.08; I^2^ = 54%), PCO₂ (MD = 3.92 [−2.84, 10.69]; P = 0.26; I^2^ = 63%), and ETCO₂ (MD = 3.51 [−5.00, 12.02]; P = 0.42; I^2^ = 94%) Supplementary Fig. 5.

Our meta-analysis showed no significant differences in physiological parameters between the TS and TT groups, including pH (MD = −0.02 [−0.05, 0.001], P = 0.10; I^2^ = 25%, 4 studies), PCO2 (MD = 6.57 [−1.35, 14.49], P = 0.10; I^2^ = 86%, 5 studies), and ETCO2 (MD = 4.36 [−3.76, 16.09], P = 0.47; I^2^ = 98%, 3 studies). The meta-analysis revealed no significant differences in physiological parameters between TS and TT, including pH (MD = −0.02 [−0.05, 0.001]; P = 0.10; I^2^ = 25%, 4 studies), PCO₂ (MD = 6.57 [−1.35, 14.49]; P = 0.10; I^2^ = 86%, 5 studies), and ETCO₂ (MD = 4.36 [−3.76, 16.09]; P = 0.47; I^2^ = 98%, 3 studies) Supplementary Fig. 6.

## Discussion

### Summary of our findings

Our meta-analysis comparing thoracoscopic and conventional TT approaches for EA/TEF repair has yielded several significant findings across operative outcomes and postoperative complications. Thoracoscopic repair was associated with a significantly longer operative time than TT (mean difference: 20.94 min), reflecting its greater technical complexity and the steep learning curve for surgeons adapting to minimally invasive techniques [38, 39]. The thoracoscopic approach showed notable advantages in several key areas. First, reduce ICU stay and shorter time to oral feeding. These improvements may be attributed to the minimally invasive nature of TS, resulting in reduced surgical trauma, less postoperative pain, and potentially faster recovery of physiological functions [40]. Second, our study showed a reduction in mortality rates. This likely reflects selective allocation of healthier, stable patients to thoracoscopic repair, while more complex or unstable cases are managed via conventional TT [41]. Third, TS was associated with fewer musculoskeletal complications, aligning with the benefits of minimally invasive surgery in preserving chest wall integrity and minimizing long-term sequelae [42, 43]. Fourth, Wound infection rates were also lower, likely due to smaller incisions and less tissue trauma [44]. However, thoracoscopic repair was associated with a higher risk of anastomotic stricture requiring dilation, possibly due to technical challenges such as restricted working space, suboptimal suturing angles, and increased anastomotic tension [45]. The learning curve associated with thoracoscopic EA/TEF repair may also contribute to this finding [39]. Our analysis revealed no notable differences between the two surgical approaches in several outcomes including anastomotic leak rates, recurrent TEF, respiratory complications, need for fundoplication, and alterations in arterial blood gases, suggesting comparable safety profiles between techniques for these specific parameters.

### Comparing our results with previous similar meta-analyses

Comparison with prior meta-analyses by Wu (2017), Way (2019), and Drevin (2021) highlights both concordant and divergent findings [6, 13, 46]. All three meta-analyses reported longer operative times with TS, reflecting its technical complexity. They also found comparable anastomotic leak rates, suggesting similar anastomotic efficacy. Wu et al. and Drevin et al. noted shorter hospital stays with TS [13, 46], while both Drevin et al. and Way et al. reported faster recovery parameters, including earlier initiation of oral feeding—findings consistent with our results [6, 13]. Reduced musculoskeletal complications, such as chest deformities, with TS align with Drevin et al.’s observations [13]. However, our observed increase in the risk of stricture associated with TS contrasts with the findings of Wu et al. and Way et al., who reported no significant difference. This discrepancy may be attributed to variations in the definitions of stricture used across the studies [6, 46]. Our finding of reduced mortality with TS contrasts with Drevin et al. [13], who reported no difference. This discrepancy underscores the need for standardized outcome measures and long-term follow-up to more clearly define the risk–benefit profile of thoracoscopic EA/TEF repair. Our study provides more recent evidence by including newer trials and a larger sample size.

### Clinical implications

The findings of our meta-analysis hold several important implications for clinical practice and surgical decision-making. The significantly reduced mortality, musculoskeletal complications, and wound infections with thoracoscopic repair suggest substantial benefits of the minimally invasive approach that extend beyond cosmesis. These benefits should be weighed against the elevated risk of anastomotic stricture requiring dilation, which represents a postoperative burden for patients and families, necessitating multiple procedures under anesthesia. The longer operative times associated with TS reflect the technical demands. Surgeons in the early phase of thoracoscopic adoption should anticipate prolonged operative times and plan accordingly [39]. Reassuringly, the absence of significant differences in pH, PCO₂, and ETCO₂ supports the physiological safety of CO₂ insufflation in neonates, addressing longstanding concerns with thoracoscopic repair [47]. Shorter ICU stays and earlier oral feeding with TS suggest potential cost-effectiveness and enhanced patient experience, potentially offsetting longer operative times. These benefits reflect broader advantages of minimally invasive surgery in pediatrics and may contribute to improved early postoperative quality of life for patients and families [48]. The absence of significant differences in anastomotic leak and recurrent TEF rates challenges earlier concerns about thoracoscopic anastomotic integrity, suggesting that with sufficient expertise, TS offers comparable technical outcomes and broadens surgical options for EA/TEF repair [49].

### Strengths and limitations

This meta-analysis is strengthened by a comprehensive search across four major databases, yielding 25 comparative studies and a large, pooled sample size, enhancing statistical power. It also evaluates a broad spectrum of clinically relevant outcomes, from perioperative metrics to post-operative complications, enabling a thorough comparison of both surgical approaches. Despite these strengths, several limitations merit consideration. First, most included studies were retrospective cohorts with only one RCT, raising potential selection bias, as TS patients may differ systematically in factors like birth weight or disease severity. Second, substantial heterogeneity—especially in operative time and length of stay—reflects variability in surgical technique and institutional protocols, limiting result interpretability and highlighting the need for standardized outcome measures. Third, the studies span multiple decades during which surgical methods evolved; early thoracoscopic cases likely represent learning curves, possibly inflating complication rates and operative times relative to current practice. The inability to consistently exclude long-gap EA across the included studies represents another limitation, as the absence of a clear cutoff to define the learning curve precludes meaningful subgroup analysis. Finally, variable follow-up durations may affect detection of long-term complications, such as recurrent strictures.

### Conclusion and recommendations

This meta-analysis of 25 comparative studies shows that thoracoscopic repair of EA/TEF offers significant benefits—reduced mortality, fewer musculoskeletal complications, lower wound infection rates, shorter ICU stays, and earlier oral feeding—compared to TT. These advantages are tempered by longer operative times and a higher incidence of anastomotic strictures requiring dilation. No significant differences were observed in anastomotic leaks, recurrent TEF, respiratory complications, or fundoplication rates between approaches. Based on these findings, we recommend thoracoscopic repair as a viable alternative to conventional TT for EA/TEF in appropriately selected patients and clinical settings where surgical expertise and resources support this approach. The decision between surgical approaches should be individualized, considering patient factors, institutional setup, and surgeon expertise. To optimize outcomes with thoracoscopic repair, we recommend structured training programs, case selection protocols that evolve with surgical experience, and standardized follow-up regimens with particular attention to early detection and management of anastomotic strictures. Future research should focus on long-term functional outcomes, quality of life measures, and strategies to decrease the risk of anastomotic stricture associated with thoracoscopic repair. Based on these findings, thoracoscopic repair is a viable alternative to conventional TT for EA/TEF in selected patients and settings with adequate surgical expertise and resources. Future research should prioritize long-term functional outcomes, quality of life, and strategies to reduce stricture risk associated with thoracoscopic repair.

## Supplementary Information

Below is the link to the electronic supplementary material.

Supplementary file1 Forest Plot of Musculoskeletal Complications (PNG 10 KB)

Supplementary file2 Forest Plot of Respiratory Complications (PNG 9 KB)

Supplementary file3 Forest Plot of Wound Infection (PNG 9 KB)

Supplementary file4 Forest Plot of Fundoplication (GERD Surgery) (PNG 13 KB)

Supplementary file5 Forest Plot of Arterial Blood Gases (Postoperative) (PNG 17 KB)

Supplementary file6 Forest Plot of Arterial Blood Gases (Intraoperative) (PNG 21 KB)

Supplementary file7 (DOCX 1138 KB)

## Data Availability

The data that support the findings of this study are available upon reasonable request.
