# Supplementary figures and images for: Thoracoscopic versus conventional thoracotomy for esophageal atresia/tracheoesophageal fistula repair: a comprehensive meta-analysis of 25 comparative studies
Source: Pediatr Surg Int. 2025 Sep 9;41(1):289. doi: 10.1007/s00383-025-06182-9 (PMC12420690; doi:10.1007/s00383-025-06182-9)

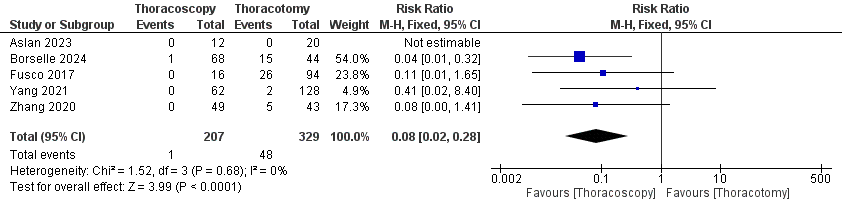

Supplement: Supplementary file 1 — Supplementary file1 Forest Plot of Musculoskeletal Complications (PNG 10 KB) [file 383_2025_6182_MOESM1_ESM.png]

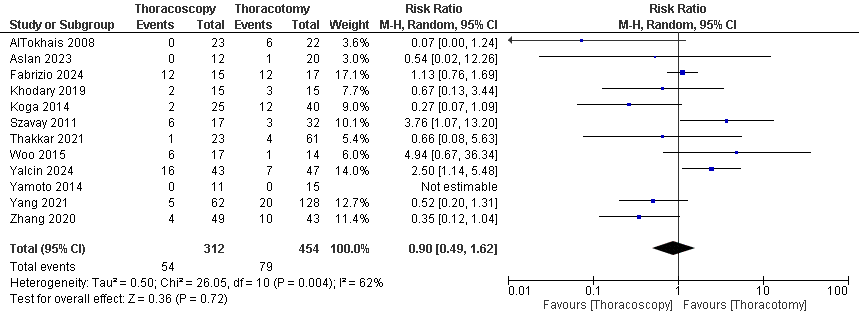

Supplement: Supplementary file 2 — Supplementary file2 Forest Plot of Respiratory Complications (PNG 9 KB) [file 383_2025_6182_MOESM2_ESM.png]

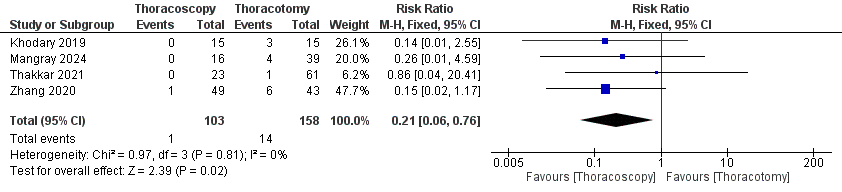

Supplement: Supplementary file 3 — Supplementary file3 Forest Plot of Wound Infection (PNG 9 KB) [file 383_2025_6182_MOESM3_ESM.png]

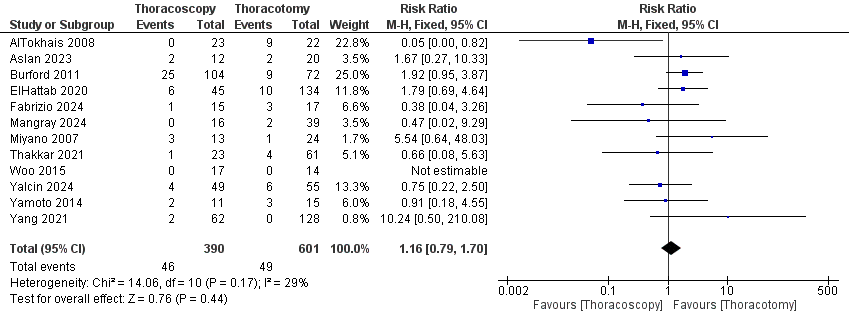

Supplement: Supplementary file 4 — Supplementary file4 Forest Plot of Fundoplication (GERD Surgery) (PNG 13 KB) [file 383_2025_6182_MOESM4_ESM.png]

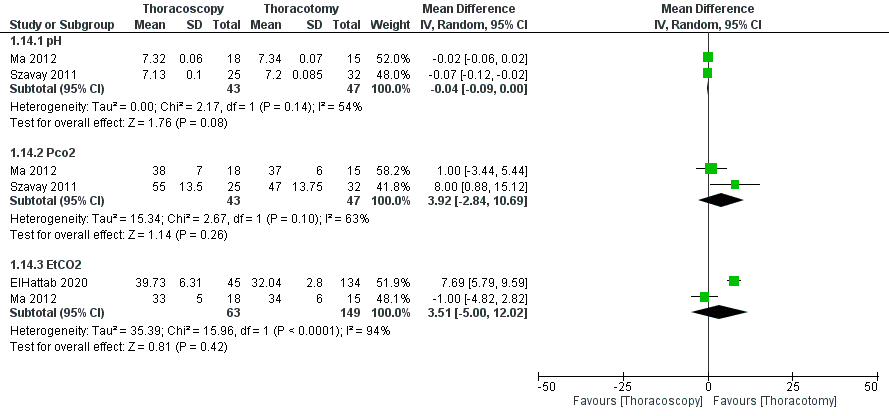

Supplement: Supplementary file 5 — Supplementary file5 Forest Plot of Arterial Blood Gases (Postoperative) (PNG 17 KB) [file 383_2025_6182_MOESM5_ESM.png]

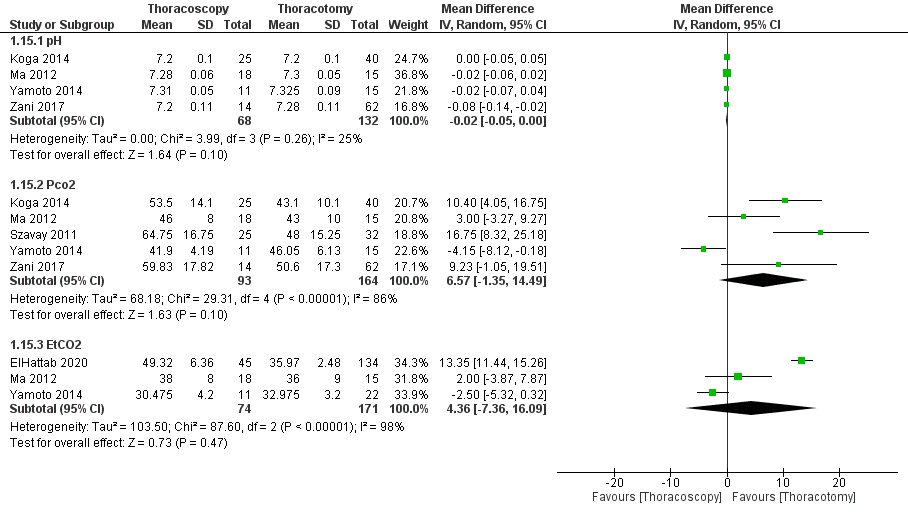

Supplement: Supplementary file 6 — Supplementary file6 Forest Plot of Arterial Blood Gases (Intraoperative) (PNG 21 KB) [file 383_2025_6182_MOESM6_ESM.png]
