# Supplementary material for: Thoracoscopic versus conventional thoracotomy for esophageal atresia/tracheoesophageal fistula repair: a comprehensive meta-analysis of 25 comparative studies
Source: Pediatr Surg Int. 2025 Sep 9;41(1):289. doi: 10.1007/s00383-025-06182-9 (PMC12420690; doi:10.1007/s00383-025-06182-9)
Supplement: Supplementary file 7 — Supplementary file7 (DOCX 1138 KB) [file 383_2025_6182_MOESM7_ESM.docx]

Table S1. Full Search Strategy Used in our search.

| Databases | Search Strategy | Date of Search |
| --- | --- | --- |
| PubMed, Web of Science (WOS), Cochrane Library, and Scopus | (“Esophag* Atresia*” OR “Esophagotracheal Fistula*” OR “Tracheoesophageal Fistula*” OR “Tracheoesophageal anomaly” OR “EA /TEF” OR “esophageal atresia with fistula” OR “Esophageal tracheal malformation” OR “foregut malformation” OR “TEF with atresia”) AND (Thoracoscopy* OR “pleura* endoscop*” OR "Minimally invasive" OR "Open surg*" OR "conventional surg*" OR "standard surg*" OR "traditional surg*" OR "classic surg*" OR thoracotomy OR Pleuroscopy OR “Video-assisted thoracoscopic surgery” OR VATS) | April 16, 2025 |

Table S2. Quality assessment of cohort studies using the NOS tool.

| ID | Selection | | | | Comparability | Outcome | | | Overall |
| --- | --- | --- | --- | --- | --- | --- | --- | --- | --- |
|  | D1 | D2 | D3 | D4 |  | D5 | D6 | D7 |  |
| **Hyman 2025** | * | * | * | * | ** | * | * | * | Good |
| **Datta 2025** | * | * | * | * | ** | * | * | * | Good |
| **Borselle 2024** | * | * | * | * | ** | * | * | * | Good |
| **Fabrizio 2024** | * | * | * | * | * | * | * | * | Good |
| **Mangray 2024** | * | * | * |  |  | * | * | * | Fair |
| **Yalcin 2024** | * | * | * | * | * | * | * | * | Good |
| **Aslan 2023** | * | * | * | * | * | * | * | * | Good |
| **Zou 2023** | * | * | * | * | ** | * | * | * | Good |
| **Thakkar 2021** | * | * | * | * | * | * | * | * | Good |
| **Yang 2021** | * | * | * | * | * | * | * | * | Good |
| **Zhang 2020** | * | * | * | * | * | * | * | * | Good |
| **ElHattab 2020** | * | * | * | * | * | * | * | * | Good |
| **Zani 2017** | * | * | * | * | ** | * | * |  | Good |
| **Fusco 2017** | * | * | * | * | ** | * | * | * | Good |
| **Nice 2016** | * | * | * | * | ** | * | * |  | Good |
| **Woo 2015** | * | * | * |  | * | * | * | * | Fair |
| **Yamoto 2014** | * | * | * | * | * | * | * | * | Good |
| **Koga 2014** | * | * | * | * | * |  | * | * | Good |
| **Ma 2012** | * | * | * | * | * | * | * |  | Good |
| **Burford 2011** | * |  | * | * |  | * | * | * | Fair |
| **Ceelie 2011** | * | * | * | * | ** | * | * | * | Good |
| **Szavay 2011** | * | * | * | * | ** | * | * | * | Good |
| **Miyano 2007** | * | * | * | * | * | * | * |  | Good |

D1: Is the case definition adequate/Representative of the exposed cohort?

D2: Representative of the cases/Selection of the non-exposed cohort.

D3: Selection of Controls/Ascertainment of exposure.

D4: Definition of Controls/ Demonstration that outcome of interest was not present at start of study.

D5: Ascertainment of exposure/ Assessment of outcome.

D6: Same method of ascertainment for cases and controls/ Was follow-up long enough for outcomes to occur.

D7: Non-Response rate/ Adequacy of follow up of cohorts.

Table S3. Quality assessment of case-control studies using the NOS tool.

| ID | Selection | | | | Comparability | Outcome | | | Overall |
| --- | --- | --- | --- | --- | --- | --- | --- | --- | --- |
|  | D1 | D2 | D3 | D4 |  | D5 | D6 | D7 |  |
| Al Tokhais 2008 | * |  | * |  | * | * | * |  | Fair |

D1) Is case definition adequate?

D2) Representativeness of the cases?

D3) Selection of Controls?

D4) Definition of Controls?

D5) Ascertainment of exposure?

D6) Same method of ascertainment for cases and controls?

D7) non-response rate?

Table S4. Quality assessment of Randomized controlled trials using the Cochrane tool.

| ID | Random sequence generation | Allocation concealment | Blinding of participants | Blinding of outcome assessment | Attrition bias | Reporting bias | Other sources of bias |
| --- | --- | --- | --- | --- | --- | --- | --- |
| Khodary 2019 | Low | Unclear | High | Unclear | Low | Low | Low |
